# Expression and clinical significance of CLDN7 and its immune-related cells in breast cancer

**DOI:** 10.1186/s13000-024-01513-1

**Published:** 2024-08-22

**Authors:** Xiaojie Fan, Aifeng Qi, Meng Zhang, Ying Jia, Shi Li, Dandan Han, Yueping Liu

**Affiliations:** 1 Departments of Pathology, the Fourth Hospital of Hebei Medical University, No.12, Jiankang Road, Shijiazhuang, 050011 PR China; 2 Shijiazhuang Hospital of Traditional Chinese Medicine, No.233, zhongshan Road, Shijiazhuang, 050011 PR China

**Keywords:** Breast cancer, Tumor immune cell infiltration, Prognosis, CLDN7, Expression

## Abstract

**Background:**

CLDN is a core component of tight junctions (TJs). Abnormal expressions of CLDNs are commonly detected in various types of tumors. CLDNs are of interest as a potential therapeutic target. CLDNs are closely associated with most cancers of epithelial origin, especially when CLDN7 promotes cancer cell metastasis, such as in gastric, cervical, and ovarian cancers.Its expression and prognosis in breast cancer (BC) remain unknown.The purpose of this study was to investigate the expression pattern of CLDN7 and related immune factors in BC and shed light on a better therapeutic avenue for BC patients.

**Method:**

The cBioPortal, GEPIA, and TCGA databases were used to comprehensively assess the expression of CLDN7 in BC. The Kaplan-Meier Plotter (KMP) database was applied to examine the relationship among the CLDN7 overexpression (OE), prognosis, and overall survival (OS) of BC patients. Immunohistochemical staining was performed on 92 BC tissue samples and 20 benign breast tumors to verify the expression level of CLDN-7 protein and its correlation with clinicopathological features and prognosis. TIMER2.0 was used to analyze the correlation between the CLDN7 OE and immune gene activation using BC-related transcriptomic data. Enrichment analyses of CLDN7-related immune pathways were conducted using online databases. The risk of expression of CLDN7-related immune genes was assessed and differentially expressed (DE) genes were included in the construction of the risk prognosis nomogram.

**Results:**

Both database analysis and clinical sample validation results showed that CLDN7 was significantly overexpressed (OE) in BC, and the OE was correlated with poor DFS in BC patients (*p* < 0.05). TIMER2.0 analysis indicated that CLDN7 OE was negatively associated with the activation of B-cells, CD4^+^ T-cells, and CD8^+^ T-cells but positively with the M_0_ macrophages. Pathway enrichment analysis suggested that CLDN7-related immune factors were mostly involved in the NF-κB and T-cell receptor (TCR) signaling pathways. Univariate Cox regression was used to analyze the correlation between 52 CLDN7 related genes and OS, and 22 genes that are related to prognosis were identified. Prognostic genes were included in the prognostic nomogram of BC with a C-index of 0.76 to predict the 3-year and 5-year OS probabilities of BC individuals.

**Conclusions:**

These findings provide evidence for the role of CLDN7-linked tumor immunity, suggesting that CLDN7 might be a potential immunotherapeutic target for BC, and its association with immune markers could shed light on the better prognosis of BC.

**Supplementary Information:**

The online version contains supplementary material available at 10.1186/s13000-024-01513-1.

## Introduction

Breast cancer (BC) is one of the most frequently diagnosed carcinomas in women worldwide, ranking second highest in terms of the mortality rate, which has been rapidly increasing every year [1]. Pathologically, BC represents a highly heterogeneous cancer subtype that varies from patient to patient. Notably, the prognosis of BC patients primarily relies on the patient’s immunity [2]. It is found that BC pathology is associated with a robust infiltration of immune-active T-cells into the lesion area, including the stroma. While the population of infiltrating CD8^+^ T-cells is significantly correlated with the immune escape capacity of cancer cells, the rate of infiltration of both CD8^+^ and CD4^+^ T-cells indicates the prognosis of the patient [3]. Importantly, macrophages contribute to roughly 50% of the invasive immune cells and are mostly responsible for promoting the anti-tumor defense mechanism [4]. Therefore, there is an urgent requirement to identify diagnostic as well as prognostic indicators to guide personalized therapy to BC patients.

Despite the recent advancement in BC diagnosis and treatment strategies, cancer recurrence and metastasis remain the major concerns for patient survival. To improve the precision of BC therapy, novel druggable targets have been discovered exploiting the factors involved in the intracellular communications and epithelial-mesenchymal transition (EMT) process. Pathological alterations in the expression of protein factors regulating various cell-cell adhesion ports, including the gap junction (GJ), tight junction (TJ), desmosomes, and adhesive junction, can induce EMT. Amongst them, abnormal expressions of several TJ-related claudins (CLDNs) are commonly detected in various types of tumors. CLDNs contain 27 families of transmembrane proteins [5]. Depending on the variations in protein sequences, CLDNs can be divided into two types, namely the classical (e.g., CLDN1-10, 14, 15, 17, and 19) and atypical (e.g., CLDN11-13, 16, 18, 20–24) [6]. CLDNs range from 20 to 34 kDa in size, consisting of N- and C-terminal cytoplasmic, four transmembrane, and two extracellular ring domains. The C-terminus of CLDN proteins are diverse in their sequences and lengths and also harbors sites (serine, threonine, and tyrosine) for the post-translational phosphorylation that regulates the protein’s structure and function. It is shown that dysregulated expression and protein phosphorylation could result in tumorigenesis and metastasis in the later stage.

Therefore, in this study, we investigated the expression pattern of CLDN7 and related immune factors in BC by bioinformatic analysis and assay to shed light on a better therapeutic avenue for BC patients.

## Materials and methods

### Downloading the database information

The cBioPortal database (https://www.cbioportal.org/) was exploited to study the CLDN7 gene mutation analysis in various types of tumors. The Human Protein Atlas (HPA) (https://www.proteinatlas.org/) was used to analysis CLDN7 cellular localization in BC cell lines (MCF-7) and expression level in BC samples. The Kaplan-Meier Plotter (KMP) (https://kmplot.com/analysis/index.php?p=service&cancer=breast) was employed to perform the correlative analyses among the CLDN7 expression profile and overall survival (OS: the period from the date of diagnosis to the date of recorded death) rate, and recurrence-free survival (RFS: the period from the date of curative surgery to the time of recurrence or death) rate in BC patients. Further, KMP was used to analyze the association between CLDN7 expression and RFS of BC molecular subtypes including Luminal A, Luminal B, HER2 enriched and TNBC. The Cancer Genome Atla (TCGA) dataset (https://portal.gdc.cancer.gov/) containing information on 1109 BC and 113 healthy controls was also used in the correlation analysis between the clinicopathological features and corresponding CLDN7 expressions. The differential expression (DE) of CLDN7 between BC and normal tissues was analyzed using the limma package (R software).

### Pathological data of clinically verified samples

Paraffin-embedded tissue samples from 92 BC and 20 benign breast tumors (controls) cases including 8 intraductal papilloma and 12 fibroadenoma were selected from the patients who received radical mastectomy (RM) surgeries in the Hebei Medical University (HMU; Fourth Hospital, Pathology department) from June 2016-17 for a standardized tissue pretreatment [7]. Follow-up data of medical records showed that these patients were never preoperatively treated with any of the radio-chemotherapy, targeted, or immunotherapy. All the above samples were obtained with informed consent and signed by the subjects or their family members. The study scheme has been reviewed and approved by the Ethics Committee of the hospital (ethics review No. 2021KY1421).

### Expression of CLDN7 in clinically verified samples

Immunohistochemical (IHC) analysis of tissue sections was performed following the EnVision method using a primary anti-CLDN7 (Abcam, ab207300, dilution 1:200) and corresponding HRP-conjugated secondary antibodies (Mixin Biological). The antibody staining was visualized by a DAB Dye solution (MaxVision; Mixin Biological). The cytoplasmic and/or cytomembrane localizations of CLDN7 were confirmed by brown-yellow staining in positive control samples. Based on the IHC staining intensities and percentages, a semi-quantitative scoring system (3 for strong, 2 for moderate, 1 for weak, and 0 for no detected expression) was adopted for assessing CLDN7 expression levels. Furthermore, proportions of CLDN7-positive tumor cells were scored as 3 for 51–100%, 2 for 11–50%, 1 for 1–10%, and 0 for no positive cell staining. Cases with a staining score of 3 (51–100%) and medium to strong (2–3) staining intensity were characterized as high expression CLDN7 patients [8]. The IHC images were captured at 100× magnification under a light microscope and scored by two experienced pathologists.

### Signal pathway enrichment

The correlation between immune gene activation and CLDN7 expression levels was analyzed by TIMER2.0 using the BC transcriptome profiles. The CLDN7-related immune modulators were explored in TISIDB, an online integrated database (http://cis.hku.hk/TISIDB/). Immuno-suppressive and stimulants genes that had been significantly correlated with the expression of CLDN7 were selected for this analysis (*P* < 0.05, Spearman’s correlation). We uploaded the CLDN7 expression regulating immunomodulators to the Cancer Genomics Student Biology (CGSB) web portal (www.cbioportal.org). By using this network module, 50 genes changed at the same time were queried. The WebGestalt toolkit (http://www.webgestalt.org/) was applied to the functional enrichment of immune modulators for the KEGG pathway analyses.

### Risk assessment

The Cox model was applied to the analysis of variables for the establishment of a prognostic risk model of BC, guided by the correlative interactions between CLDN7 and other immune-associated genes. After the initial selection, the prognostic index (risk-score) was derived using the formula: risk-score = a1b1 + a2b2+… + aibi, where a1 refers to the expression level of individual genes, and b1 is the risk coefficient of each gene from the Cox model. The KM survival curve, log-rank test, and Cox analysis were employed to evaluate the relationship among immune-related gene characteristics, clinical features, and OS. In this analysis, we included the factors like gender, age, cancer staging (TNM staging), and other related parameters. The R language ROC software package was used to assess the accuracy of the risk-score.

### Line graph construction

The prognosis of BC patients was assessed by combining the patient’s risk-score with the clinical characteristics. A standard calibration curve was constructed to visualize the deviation of the predicted probability from the observed one for individual patients. The consistency index (C-index) indicated the predictive accuracy of the nomogram.

### Statistical analysis

SPSS v26.0 and R v4.0.2 software packages were used for the data analysis and GraphPad Prism 9 was used for plotting the graphs. Measurement data were presented as ‾χ ± s, and between-group comparisons were performed using two independent sample t-tests. Count data were expressed as percentages and cases. The patients’ survival curves were constructed by the KM method, while the survival rates of the patients were compared by the log-rank test. Any statistical correlations between the CLDN7 expression level and clinicopathologic features of BC patients were determined by the chi-squared (χ^2^) test. Spearman’s correlation analysis indicated if there was any correlation between the population of tumor-infiltrating immune cells and the modulation of CLDN7 expression. *P* < 0.05 was considered the threshold for any statistically significant differences.

## Results

### Altered expressions of CLDN7 in BC tissues

**Fig. 1:**
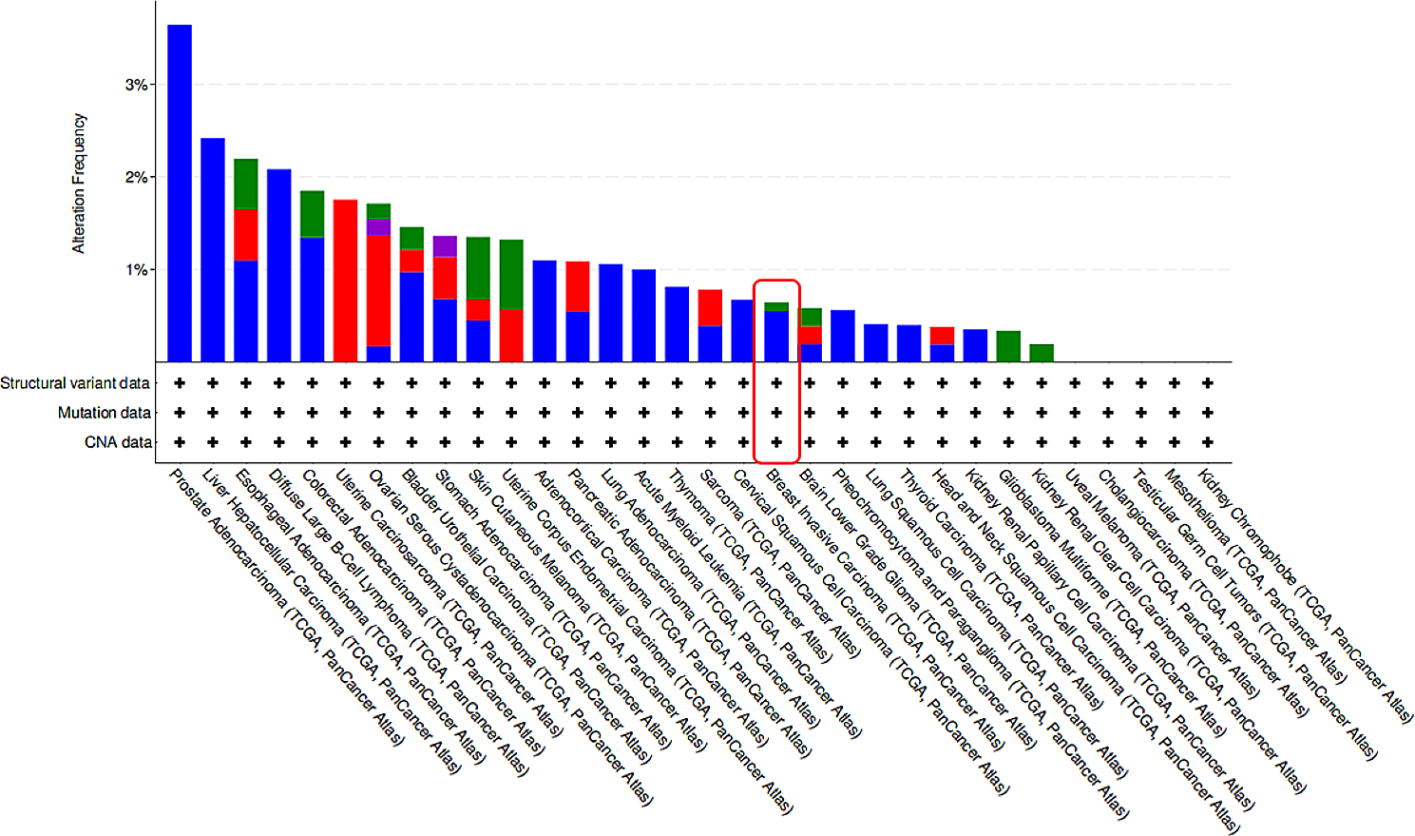
Mutation, amplification and deletion of CLDN7 in multiple cancers from cBioPortal tumor database

**Fig. 2:**
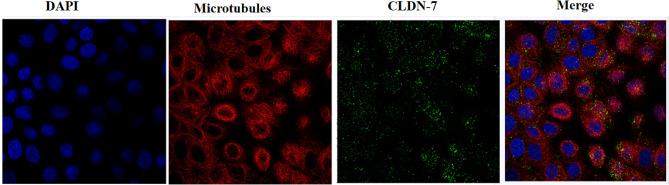
Immunofluorescence detection of CLDN7 in MCF-7 breast cancer cells from The Human Protein Atlas database

**Fig. 3:**
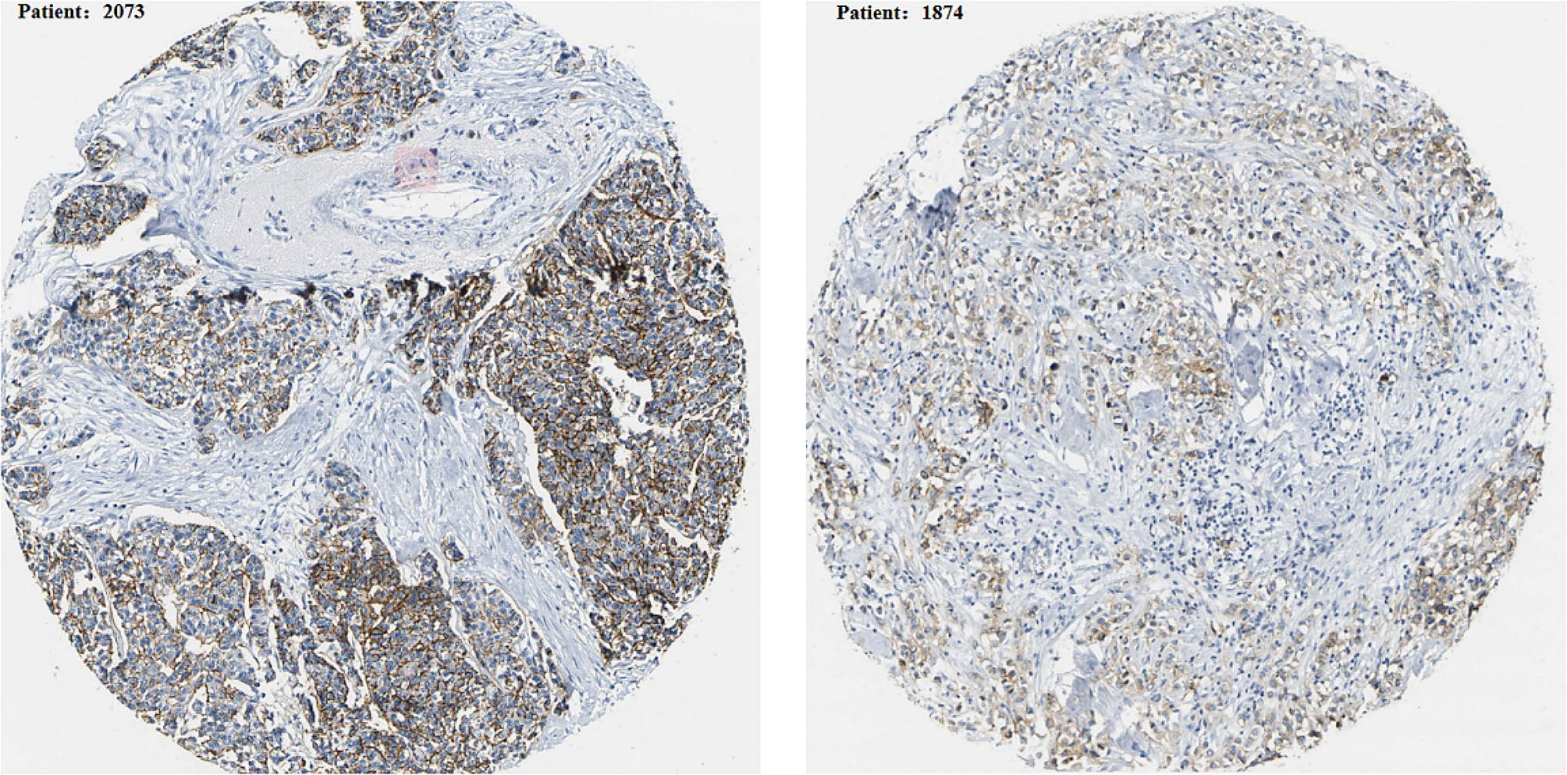
Expression of CLDN7 in breast cancer tissues from The Human Protein Atlas database

Analysis of the cBioPortal tumor database showed that the *CLDN7* gene could be mutated, downregulated, and even deleted in BC and many other cancers (Fig. 1). Consistent with the HPA database, immunofluorescence (IF) results of the CLDN7’s subcellular localization indicated its preferential abundance in the cytoplasmic membranes and/or cytomembrane in the MCF-7 cells (Fig. 2). Furthermore, IHC analysis confirmed overexpression of CLDN7 in BC tissues (Fig. 3).

### Correlation between the CLDN7 expression and prognosis of BC

**Fig. 4:**
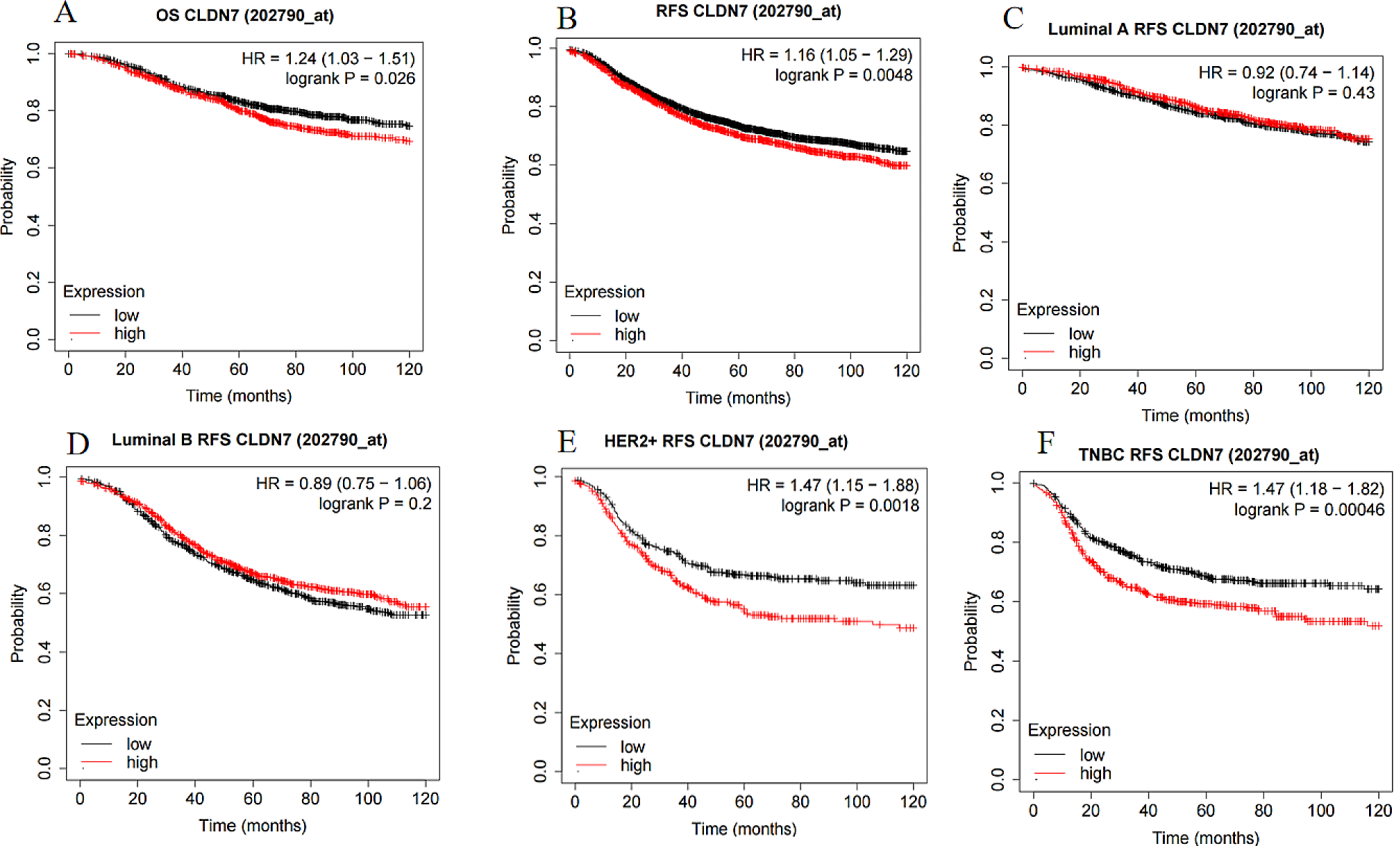
The relationship between the expression level of CLDN7 in breast cancer from Kaplan-Meier Plotter database.**(A-B)** highly expressed CLDN7 displayed shorter **(A)** OS, and **(B)**RFS time. **(C-F)** Subtype analysis showed that **(C)** luminal A, **(D)** luminal B, **(E)** HER2 enriched, and **(F)**TNBC

The KMP analysis revealed that the BC patients with CLDN7 overexpression (OE) had poor OS than those with the CLDN7 low expression profile (HR = 1.24, *P* = 0.026; Fig. 4A). Further analysis of the RFS of BC patients exhibited significantly reduced rates in cases with the CLDN7 OE than those with the low expression of CLDN7(HR = 1.16,*P* = 0.0048; Fig. 4B). Correlation analysis between the expression level of CLDN7 and survival prognosis by molecular subtypes showed no correlation between the CLDN7 expression modulation and the RFS rates of Luminal A (HR = 0.92, *P* = 0.43; Fig. 4C) and Luminal B (HR = 0.89, *P* = 0.2; Fig. 4D). The rate of RFS was significantly lower in BC patients overexpressing both HER2 and CLDN7 compared to their low-expression counterparts (HR = 1.47,*P* = 0.0018; Fig. 4E). Notably, the RFS of the triple-negative BC (TNBC) subtype with the CLDN7 OE was significantly lower than the respective controls (HR = 1.47, *P* = 0.00046; Fig. 4F).

### Correlation between CLDN7 expression and pathological features in BC tissues of TCGA database

**Fig. 5:**
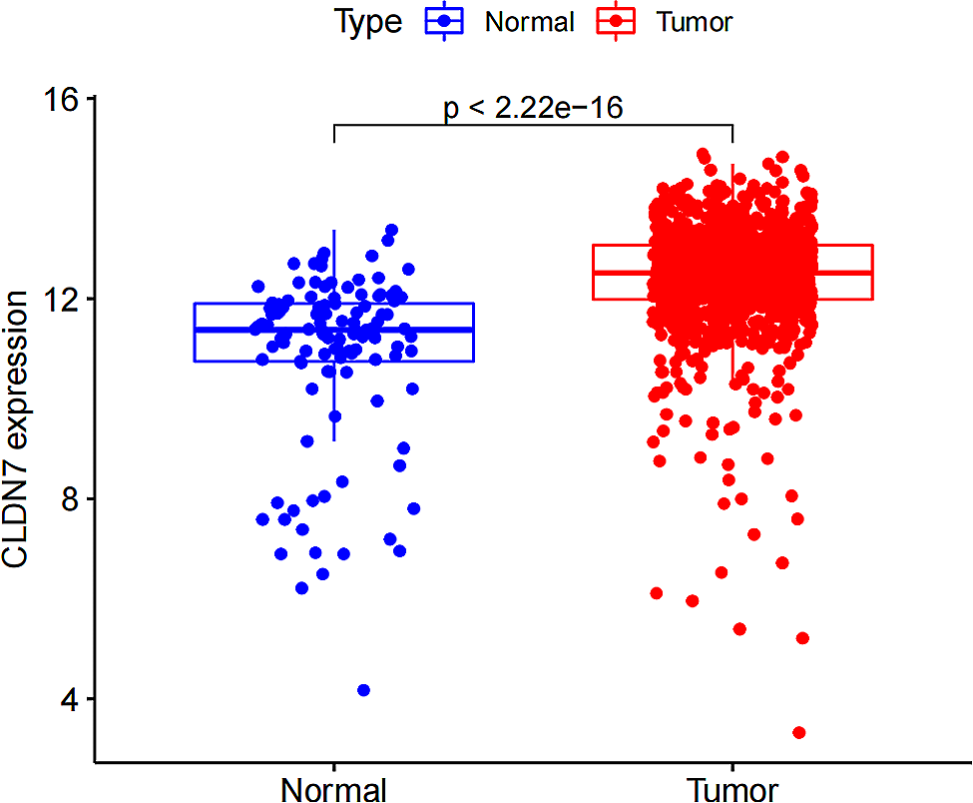
Expression of CLDN7 in normal and tumor of breast tissues

TCGA-BC transcriptomic analysis using the limma package showed significant differences in CLDN7 expressions between the BC and control tissues (*P* < 0.05; Fig. 5). Moreover, correlation analysis between the CLDN7 expression and pathological features in 908 BC samples (TCGA) with complete clinical information revealed that the altered expression of CLDN7 could be associated with expressions of the estrogen receptor coding gene *ESR1* and the human epidermal growth factor receptor encoding gene *ERBB2* (All *P* < 0.05; Table 1).

**Table 1:** TCGA queue CLDN7 correlation with clinical pathological characteristics [n (%)]

Clinical Features	Total	CLDN7 high expression	CLDN7 low expression	χ2	*P*
	(*N* = 908)	(*N* = 366)	(*N* = 542)		
Age/year				3.059	0.08
<60	499(54.96)	214(58.47)	285(52.58)		
≥ 60	409(45.04)	152(41.53)	257(47.42)		
Gender					
Female	897(98.79)	363(99.18)	534(98.52)	0.826	0.363
Male	11(1.21)	3(0.82)	8(1.48)		
AJCC Stage				3.287	0.349
I	160(17.62)	57(15.57)	103(19.00)		
II	532(58.59)	214(58.47)	318(58.67)		
III	199(21.92)	86(23.50)	113(20.85)		
IV	17(1.87)	9(2.46)	8(1.48)		
T				5.657	0.13
T1	236(25.99)	145(39.62)	91(16.48)		
T2	538(59.25)	326(89.07)	212(39.11)		
T3	102(11.23)	58(15.85)	44(8.12)		
T4	32(3.52)	13(3.55)	19(3.51)		
N				2.073	0.557
N0	450(49.56)	171(46.72)	279(51.48)		
N1	301(33.15)	127(34.70)	174(32.10)		
N2	103(11.34)	44(12.02)	59(10.89)		
N3	53(5.84)	24(6.56)	30(5.54)		
M				1.149	0.284
M0	891(98.13)	357(97.54)	534(98.52)		
M1	17(1.87)	9(2.46)	8(1.48)		
ESR1				8.578	0.003
+	340(37.44)	158(43.17)	182(33.58)		
-	568(62.56)	208(56.83)	360(66.42)		
ERBB2					
+	14(15.75)	76(20.77)	67(12.36)	11.627	0.001
-	765(84.25)	290(79.23)	475(87.64)		
PGR				0.045	0.833
+	257(28.30)	105(28.69)	152(28.04)		
-	651(71.70)	261(71.31)	390(71.96)		

### Expression of CLDN7 in clinical samples

**Fig. 6:**
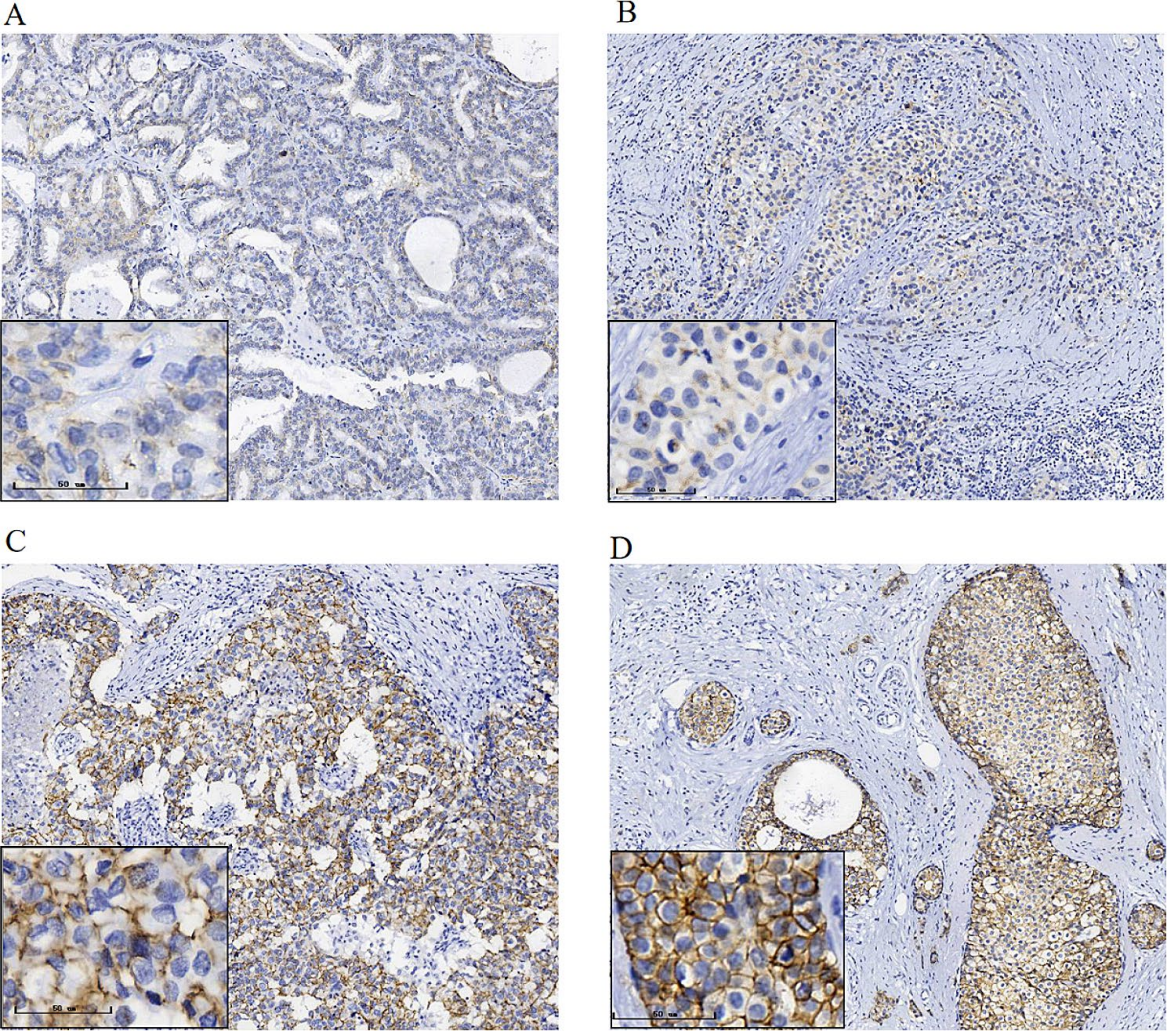
Expression of CLDN7 in benign of the breast and breast cancer tissue detected by IHC (larger map, x100; small icon, x400). **(A)**Weak staining for CLDN7 in benign breast tumor **(B)**Weak staining for CLDN7 in BC **(C)** Moderate staining for CLDN7 in BC **(D)** Strong staining for CLDN7 in BC

**Fig. 7:**
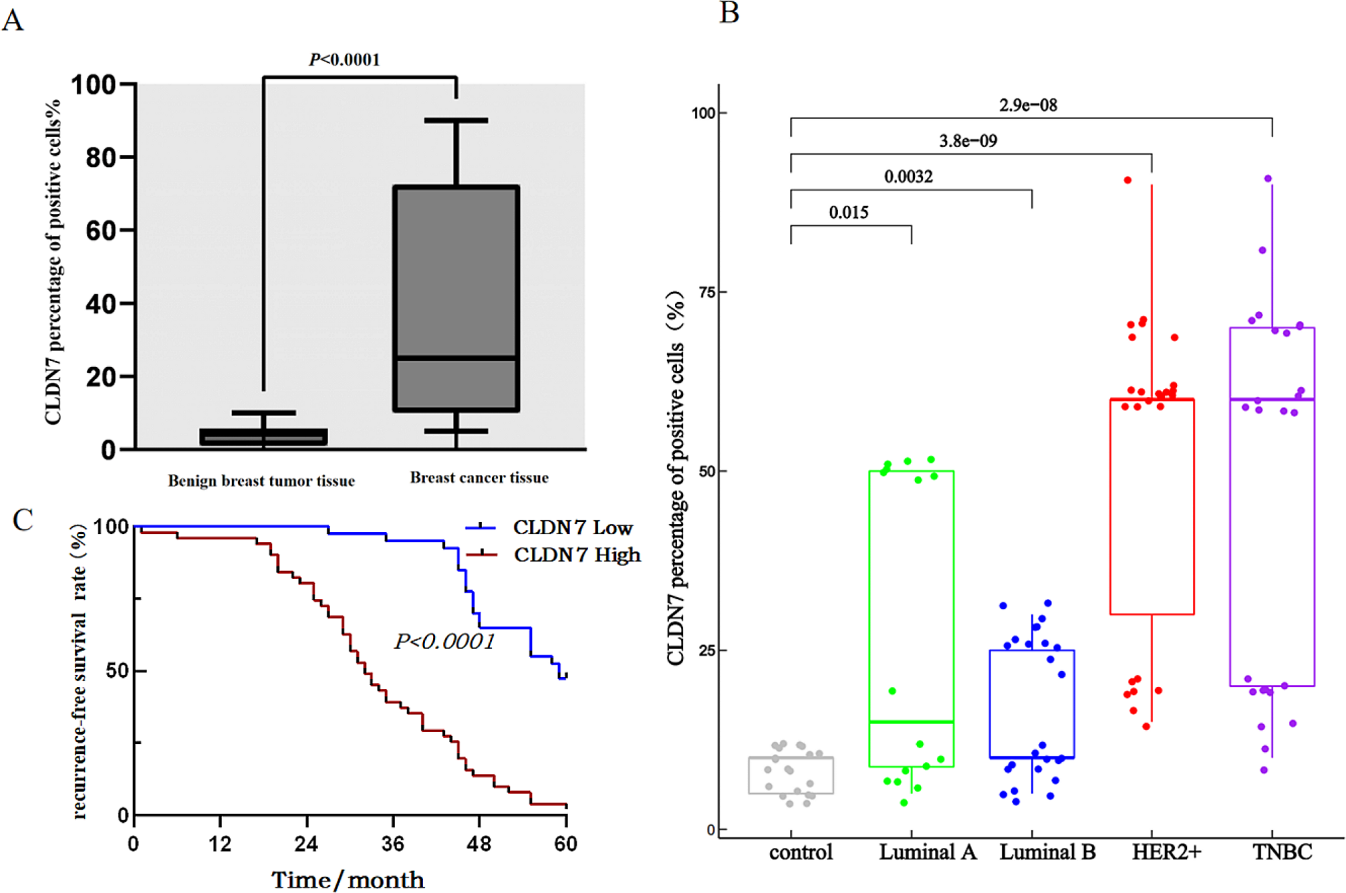
Expression level of CLDN7 in breast cancer tissues and its relationship with patients’ RFS. **(A)** Quantitative analysis of CLDN7 in benign breast tumor tissue and breast cancer tissue **(B)** Expression levels of CLDN7 in subtypes of breast cancer **(C)** CLDN7 expression and RFS analysis in breast cancer patients

Pathological expressions of CLDN7 in 92 clinically confirmed BC samples are shown in Table 2. CLDN7 levels were significantly higher in BC samples than that in the benign tumor samples (*P* < 0.05; Fig. 6). Besides, almost all subtypes of cancer cells exhibited CLDN7 OE-positive cells than that in the control samples (*P* < 0.05; Fig. 7A-B). The KM survival analysis further verified that the RFS was drastically reduced in BC patients with CLDN7 OE (*P* < 0.05;Fig. 7C).

**Table 2:** Correlation between CLDN7 and clinicopathological features in clinically confirmed BC samples [n (%)]

Clinicopathological features	Total	CLDN7 high expression	CLDN7 low expression	χ2	*P*
	(*N* = 92)	(*N* = 52)	(*N* = 40)		
Age/year				0.031	0.859
<60	63(68.48)	36(69.23)	27(67.50)		
≥ 60	29(31.52)	16(30.77)	13(32.50)		
Histological grade				0.117	0.943
I	29(31.52)	16(30.77)	13(32.50)		
II	36(39.13)	20(38.46)	16(40.00)		
III	27(29.35)	16(30.77)	11(27.50)		
Clinical stage				0.449	0.799
I	26(28.26)	14(26.92)	12(30.00)		
II	40(43.48)	22(42.31)	18(45.00)		
III	26(28.26)	16(30.77)	10(25.00)		
Tumor size(cm)				0.054	0.816
<2	61(66.30)	35(67.31)	26(65.00)		
≥ 2	31(33.70)	17(32.69)	14(35.00)		
Lymph node metastasis				6.988	0.008
N0	50(54.35)	22(42.31)	28(70.00)		
N1 + N2 + N3	42(45.65)	30(57.69)	12(30.00)		
Distant metastasis				6.776	0.009
M0	68(73.91)	33(63.46)	35(87.50)		
M1	24(26.09)	19(36.54)	5(12.50)		
ER				0.033	0.855
-	47(51.09)	27(51.92)	20(50.00)		
+	45(48.91)	25(48.08)	20(50.00)		
HER-2				5.12	0.024
-	62(67.39)	30(57.69)	32(80.00)		
+	30(32.61)	22(42.31)	8(20.00)		
PR				0.097	0.755
-	50(54.35)	29(55.77)	21(52.50)		
+	42(45.65)	23(44.23)	19(47.50)		
Type of molecule				5.68	0.128
Luminal A	16(17.39)	7(13.46)	9(22.50)		
Luminal B	25(27.17)	11(21.15)	14(35.00)		
HER2 enriched	26(28.26)	19(36.54)	7(17.50)		
TNBC	25(27.17)	15(28.85)	10(25.00)		

### Correlation between CLDN7 expression and immune cell activation

**Fig. 8:**
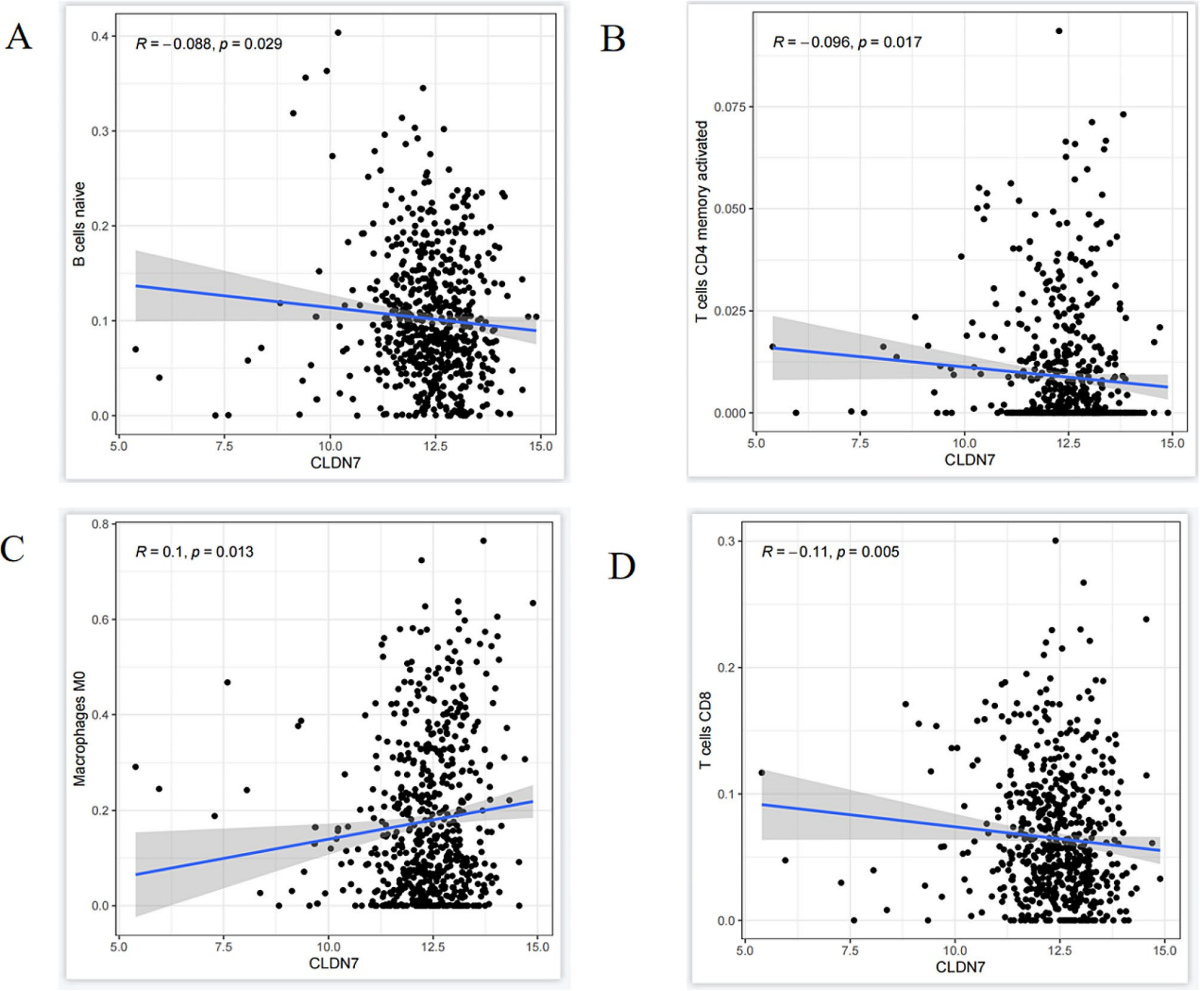
Correlation between CLDN7 expression and immune cell infiltration in breast cancer tissues. **(A)** CLDN7 expression was correlated with B cells **(B)** CLDN7 expression was correlated with CD4 + T cells **(C)** CLDN7 expression is correlated with M0 macrophages **(D)** Correlation between CLDN7 expression and CD8 + T cells (Pearson correlation test)

The TIMER2.0 analysis revealed that the rate of immune cell infiltration into the tumor was increased in association with CLDN7 OE in BC samples than in the controls (Fig. 8). The CLDN7 OE was negatively correlated with the activation of B-cells, and CD4^+^/CD8^+^ T-cells but positively with the M_0_ macrophages.

### Enrichment analyses

**Fig. 9:**
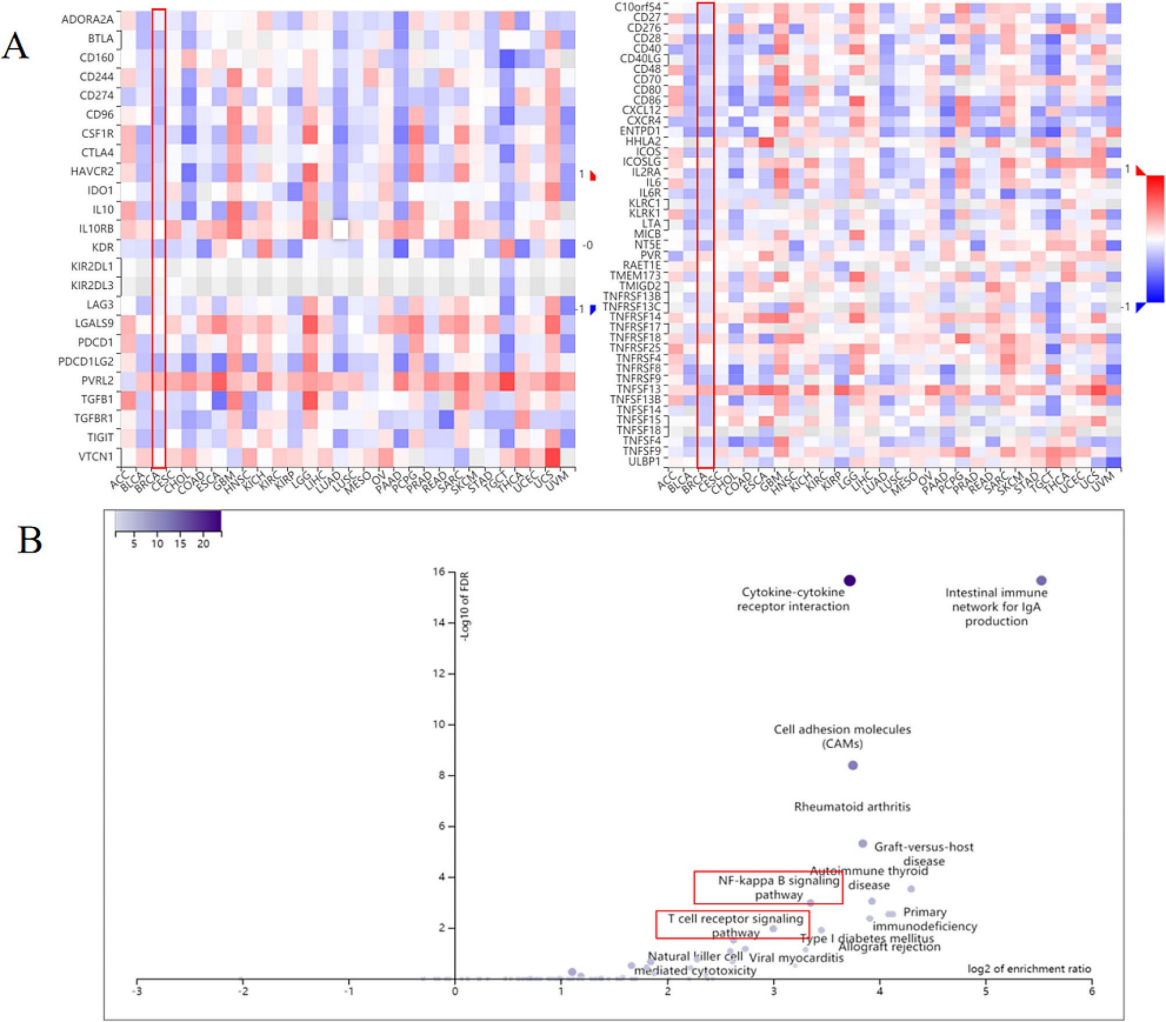
Identification and analysis of CLDN7 gene-related immunomodulators. (**A**) Heat map of immunosuppressive gene correlation with CLDN7 gene in breast cancer (left) ; Heat map of immunostimulatory gene correlation with CLDN7 gene in breast cancer (right) **(B)** Enrichment of CLDN7-related immunomodulatory genes and the signaling pathways involved in 50 closely related genes

Analysis of the TISIDB identified CLDN7-associated 15 immunosuppressive (*BTLA, CD274, CD244, CD160, CD96, CSF1R, CTLA4, KDR, HAVCR2, IDO1, PDCD1, PDCD1LG2, TIGIT, PVRL2 and TGFBR1*) and 37 immunostimulatory (*C10ORF54, CD80, CD70, CD48, CD27, CD86, CD28, CD40LG, ENTPD1, CXCL12, CXCR4, ICOSLG, ICOS, IL2RA, IL6, IL6R, KLRK1, KLRC1, LTA, MICB, NT5E, PVR, TMEM173, TNFSF4, TNFRSF8, TNFRSF14, TNFRSF9, TNFRSF13B, TNFRSF13C, TNFSF14, TNFRSF17, TNFRSF18, TNFSF9, TNFSF13, TNFSF13B, ULBP1 and TNFSF15*) genes(Fig. 9A). The top 50 genes associated with these immunomodulatory functions were analyzed using cBioPortal. The KEGG analysis revealed that these genes were mainly enriched in the NF-κB and T-cell receptor (TCR-mediated)signaling pathways (Fig. 9B).

### Assessment of the prognostic value of CLDN7-related immunomodulators in BC

**Fig. 10:**
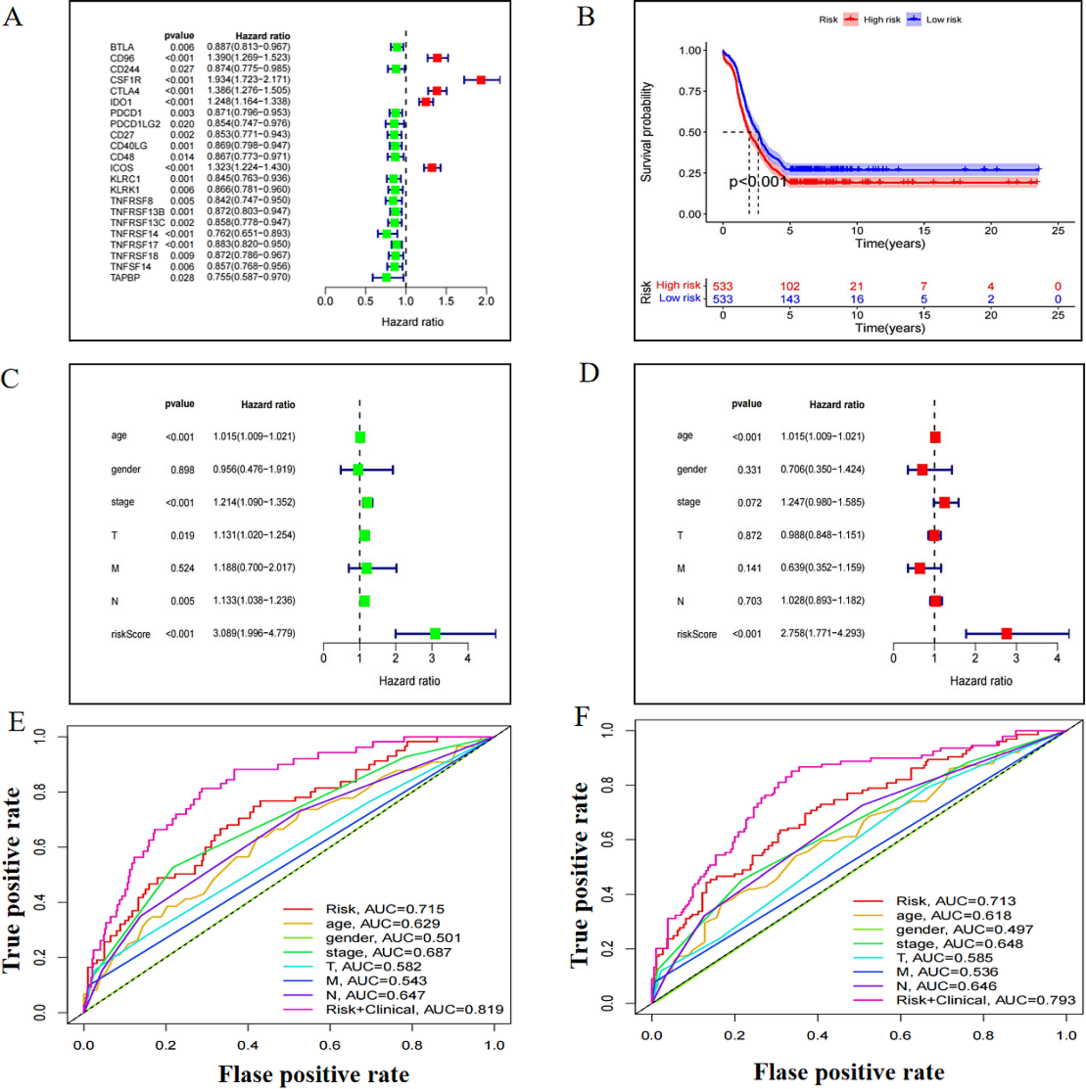
Prognostic value of CLDN-7-related immunomodulatory genes in breast cancer. **(A)** Forest map of risk scores of genes associated with breast cancer prognosis **(B)** Kaplan-Meier survival curve of prognosis model constructed by risk score **(C)** A univariate Cox regression analysis of risk scores for overall survival in the show **(D)** Multivariate Cox regression analysis of risk scores for breast cancer with respect to overall survival **(E)** 3-year ROC curve of breast cancer risk score **(F)** 5-year ROC curve of breast cancer risk score

Univariate Cox regression analysis of the correlation between the expression of immunomodulatory genes and the OS in 1109 BC and 113 controls (TCGA) identified 22 genes, including 5 high-risk and 17 low-risk, that could have significant prognostic implications to BC pathogenesis (Fig. 10A). Furthermore, a multivariate Cox regression analysis of risk-scores was used for the construction of the prognostic model. The KM survival curve indicated that low-risk-score subjects had longer survivals than those with high-risk-scores (*P* < 0.001; Fig. 10B). As shown in Fig. 10C, the risk-score had a significant correlation with the OS in BC patients in the univariate Cox regression model (HR = 3.089; 95%CI = 1.996–4.779; *P* < 0.001). Consistently, the multivariate Cox regression model showed a similar trend in BC survival (HR = 2.758; 95%CI = 1.771–4.293; *P* < 0.001; Fig. 10D). Furthermore, the AUC of the risk-score for predicting 3-year OS in BC was 0.715, and the risk-score plus clinical feature for predicting 3-year OS in BC was 0.819 (Fig. 10E). Likewise, the AUC of the risk-score for predicting 5-year OS in BC was 0.713, and that for the risk-score plus clinical feature was 0.793 (Fig. 10F).

### Line graph construction

**Fig. 11:**
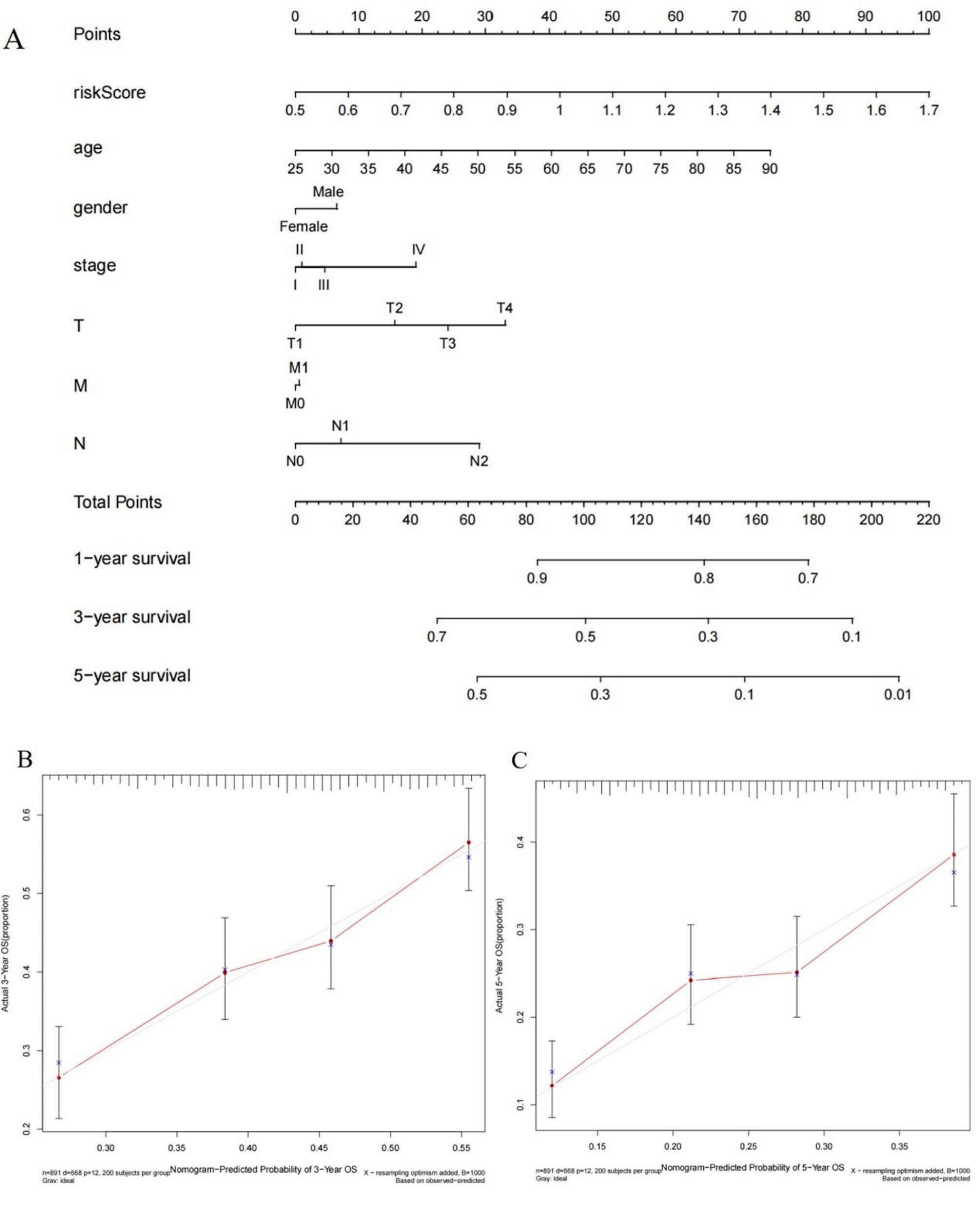
Prognosis histogram of breast cancer (**A**) Histogram of 1-year, 3-year, and 5-year survival probability of individual breast cancer patients **(B)** 3-year calibration curve for breast cancer patients **(C)** Calibration curve for 5-year survival of breast cancer patients

Based on the results of Cox regression, a prognosis histogram of BC was constructed to predict individual survival probability by weighing the risk-score, gender, age, clinical staging, and TNM staging (Fig. 11A). The calibration curve was used to calibrate the line graph, which established a relationship between the probability of survival rate predicted by the line graph (solid line) and the concept reference line (dashed line) (Fig. 11B-C). The dashed line at 45 °C represents the perfect agreement between the line graph prediction and the true probability. By using the C-index to assess the predictive differential power of the line graph, the experiment showed agreement between the prediction probability of the histogram and the actual observation of death. The C-index of the prognosis line graph was 0.85.

## Discussion

BC remains the most frequently diagnosed malignancy in women. Several high-efficiency antibodies targeting the immune checkpoints in BC cells were approved by FDA [9–11]for treating BC. However, complete eradication of carcinoma by tumor-specific T-cells involves a highly orchestrated and complex mechanism [12], and the efficacy of immunotherapy majorly depends on the successful completion of each in the whole process. As a result, it’s crucial to develop therapeutically more effective drug design as well as a precise screening of suitable BC patients for the intended treatment. However, precision biomarkers, capable of indicating a patient’s immune status and prognosis, would be of great value in improving treatment decisions for BC patients.

CLDNs, together with occlusion proteins and cell adhesion molecules comprise a tightly connected transmembrane complex that functions as a paracellular barrier and intracellular signaling port in regulating the proliferation, differentiation, and apoptosis of epithelial cells [13]. It’s shown that CLDN is closely associated with most cancers of epithelial origin, especially when CLDN7 promotes cancer cell metastasis, such as in gastric, cervical, and ovarian cancers [14–16]. However, the CLDN1 OE is shown to induce epithelial-to-mesenchymal metastasis, while CLDN7 acts as a tumor suppressor in colorectal cancer [17]. Thus, CLDN7 can simultaneously play both tumor-promoting and inhibitory roles in human malignancies, depending on the type of tumor.

In this study, cBioPortal, HPA, and KMP databases were employed to analyze the CLDN7 expression profile in BC tissues, which showed a significant upregulation in CLDN7 expression and correlated with adverse effects on OS and DFS rates in BC. The TCGA-BC cohort study has demonstrated that CLDN7 OE is strongly correlated with that of ESR1 and ERBB2. Further analysis of clinical samples revealed that the CLDN7 protein level was correlated with the HER2 level and lymph node as well as metastasis and distant metastases. HER2 (ERBB2, neu) status assessment is routinely used in the molecular diagnosis of BC. Analysis of a large cohort of BC patients has found that HER2 OE could be associated with poor prognosis and poor response to chemotherapy [18]. Notably, lymph node metastasis is the most critical indicator of OS and DFS in BC patients. Accurate evaluation of lymph node involvement is an important component of BC staging [19]. Moreover, EMT plays an important role in promoting distant metastasis and tumor infiltration in BC [20]. Therefore, it is speculated that CLDN7 may have a pro-tumor role in BC pathogenesis.

Additionally, we used the TIMER2.0 database to explore the correlative relationship between the CLDN7 OE and immune cell infiltration in BC. We found that the CLDN7 OE was negatively associated with the activation of B-cells and CD4^+^/CD8^+^ T-cells but positively with the abundance of the M_0_ macrophages. Analyses of the TISIDB and cBioPortal indicated that CLDN7-related expression of immunomodulatory genes was mainly enriched in the NF-κB and TCR signaling pathways. Corresponding studies have found that CLDN7 is involved in the inflammatory responses through NF-κB signaling, and the addition of NF-κB inhibitors can inhibit the increase of CLDN7 level [21, 22]. The NF-κB signaling is involved in tumor angiogenesis and the transcriptional activation of tumorigenic chemokines. Thus, hyperactivation of the NF-κB signaling could cause dysregulated expression of several immune-modulatory factors, including cytokines, chemokines, adhesion factors, and inhibitors of apoptosis [23]. Hence, the role of NF-κB signaling in immunotherapy has been extensively investiagated [24–26]. An NF-κB inhibitor (DHMEQ) has demonstrated effective inhibition of both early and late-stage metastases [27, 28]. Inhibitors and antibodies of the NF-κB signaling pathway factors have been targeted to neutralize immune checkpoints in clinical trials of lung cancer [24]. In summary, CLDN7 inhibitors can be predicted biologically.

The opening up of high-throughput gene expression datasets has immensely contributed to the discovery of potential biomarkers of BC to improve the prognosis [29–31]. A genome-wide gene expression profile analysis of 6,415 BC patients’ samples from 22 public cohorts has established an immune-related prognostic scoring (IRPS) system to correlate the OS outcomes with immunophenotypic factors in BC patients. Chemotherapy has been shown to increase the IRPS, indicating that chemotherapeutics can stimulate the immune signal spectrum in BC patients, and is sufficient to predict a patient’s response to the immune-checkpoint inhibitors [32]. Han et al. have analyzed the gene expression profiles of BC samples through cancer gene mapping and in vitro studies [33] showing that DEs of immune-related genes are closely associated with BC recurrence. The study has also constructed prognostic signatures for eight immune genes that might be involved in increasing the risk of relapse in subgroups of BC patients [33]. Similarly, our study established an immune genetic profile of BC using CLDN7-related immunomodulators. Risk-scores derived from genetic traits were significantly associated with the OS rate in BC patients. Most immune genes, integrated into the prognostic signals, are involved in the regulation of T-cell activation, highlighting the importance of T-cell-mediated immunity in CLDN7 overexpressing BC patients. Finally, we constructed a personalized prognostic column graph with a C-index of 0.85. Our results suggest that risk-score analysis from the DE profile of CLDN7-related immunomodulatory genes can stratify the risk groups, and the discovery of intuitive expression of CLDN7 in BC tissues may improve the validation of BC prognosis.

In conclusion, these results suggest that CLDN7 may play a critical role in the regulation of tumor immune-microenvironment. Prognostic signals from the CLDN7-related immunomodulators can independently predict the OS rate in BC. More prospective studies would require to further validate the clinical application of this prognostic biomarker in the personalized therapy of BC.

### Electronic supplementary material

Below is the link to the electronic supplementary material.

Supplementary Material 1

Supplementary Material 2

## Data Availability

No datasets were generated or analysed during the current study.

## Abbreviations

HPA: Human Protein Atlas
KMP: Kaplan-Meier Plotter
OS: Overall Survival
RFS: Recurrence-Free Survival
TCGA: The Cancer Genome Atla
DE: Differential Expression
IHC: Immunohistochemical
CGSB: Cancer Genomics Student Biology
